# CARGO: A Cytometry Analysis framework via Regularized Graph Optimal-transport

**DOI:** 10.1371/journal.pcbi.1014358

**Published:** 2026-06-23

**Authors:** Abida Sanjana Shemonti, Grzegorz B. Gmyrek, Katrien L. A. Quintelier, Sofie Van Gassen, Yvan Saeys, Marcella Willemsen, Joachim G. J. V. Aerts, Eva V. E. Madsen, J. Paul Robinson, Alex Pothen, Bartek Rajwa

**Affiliations:** 1 Department of Computer Science, Purdue University, West Lafayette, Indiana, United States of America; 2 Miftek Corporation, West Lafayette, Indiana, United States of America; 3 Department of Pulmonary Medicine, Erasmus Medical Center, Rotterdam, Zuid-Holland, The Netherlands; 4 Data Mining and Modeling for Biomedicine, VIB Center for Inflammation Research, Ghent, Belgium; 5 Department of Mathematics, Computer Science and Statistics, Ghent University, Ghent, Belgium; 6 Department of Surgical Oncology, Erasmus Medical Center, Rotterdam, The Netherlands; 7 Department of Basic Medical Science, College of Veterinary Medicine & Weldon School of Biomedical Engineering, Purdue University, West Lafayette, Indiana, United States of America; 8 Bindley Bioscience Center, Purdue University, West Lafayette, Indiana, United States of America; University Hospital Schleswig-Holstein - Campus Kiel: Universitatsklinikum Schleswig-Holstein, GERMANY

## Abstract

Conventional data visualization techniques in single-cell analysis (such as two-dimensional dot plots, SPADE, PCA, t-SNE, or UMAP) often fall short in enabling an intuitive understanding of high-parameter flow cytometry data. These methods tend to oversimplify complex biological relationships, lack biologically meaningful interpretations, and offer no principled framework for downstream quantitative analysis. To address these limitations, we present a graph-based (network-based) visualization framework grounded in optimal transport theory. In this framework, cell populations are defined by their marker-expression profiles, and inter-population similarity is quantified using an efficiently computable optimal transport formulation known as the Sinkhorn distance. Our approach produces biologically consistent two-dimensional graph layouts using a phenotype-aware Hamming distance. Structural differences between sample graphs are characterized through a customized graph-edit distance that captures changes in population size, marker expression, and relationships between populations. We demonstrate our methods on two flow cytometry datasets: one from a clinical trial of dendritic cell-based immunotherapy in malignant peritoneal mesothelioma, involving 14 patients sampled at three time points with 14-color panels, and another from FlowCAP-II, which involved 43 acute myeloid leukemia patient samples analyzed with 7-color panels. Our framework produces robust, quantitative visual summaries of cell populations and supports statistical analysis based on graph edit distances, thereby offering new insights into disease progression and treatment response. Ultimately, our method bridges the gap between flow cytometry data visualization and biological interpretation.

## Author summary

Understanding the immune system requires analyzing millions of cells, each carrying unique biological signals. Traditional visualization tools, like scatter plots or popular algorithms such as t-SNE and UMAP, help simplify this complexity but often distort important relationships between cell types. In this work, we introduce a new way to visualize flow cytometry data that is both mathematically rigorous and easy to interpret. Our method uses a concept called “optimal transport” to measure how similar or different two cell populations are and represents these relationships as a graph (network). This graph shows how immune cells interact and how these interactions change over time or between patients. We applied this approach to cancer immunotherapy and leukemia datasets, uncovering patterns that were hard to detect with existing tools. By providing clear visual summaries and quantitative comparisons, our framework can help researchers and clinicians track treatment responses and identify early signs of disease progression, insights that could ultimately guide more personalized and effective therapies.

## 1. Introduction

Flow cytometry (FC) is a high-throughput, multiparameter optical detection technology used to analyze the physical, biochemical, and functional properties of cells in a fluid suspension. By measuring thousands of cells per second, FC yields detailed information on cell size, internal complexity, and the presence of surface or intracellular markers (typically proteins) that define cell identity, activation state, and lineage. Modern instruments can assess up to 50 markers simultaneously [1], enabling comprehensive profiling of multiple biological phenotypes in a single experiment. However, conventional two-dimensional dot plots in marker space are inadequate to capture and represent the full complexity of these high-dimensional datasets.

**Fig 1:**
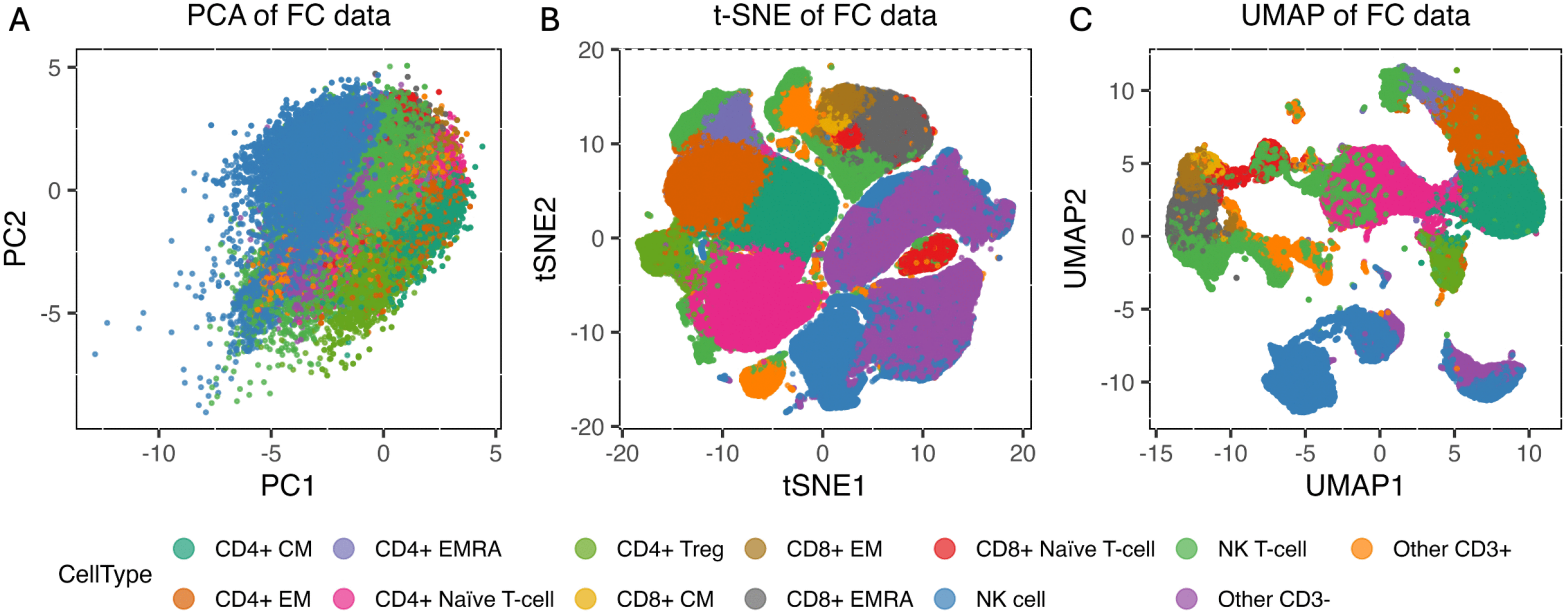
Three dimensionality reduction methods for flow cytometry data illustrating natural killer (NK) cells and T-cell populations. **(A)** PCA. **(B)** t-SNE. **(C)** UMAP. The plots are generated using the default set of parameters and the same flow cytometry sample (baseline sample of patient MCV005 in the malignant peritoneal mesothelioma dataset).

To address this limitation, the FC research community employs a range of computational approaches that underpin modern analysis and visualization pipelines. These can be broadly grouped into three categories: (a) *visualization following dimensionality reduction* techniques, including approaches such as principal component analysis (PCA) and multidimensional scaling (MDS), as well as nonlinear manifold-learning methods such as t-SNE (t-distributed Stochastic Neighbor Embedding) [2], UMAP (Uniform Manifold Approximation and Projection) [3], and PHATE (Potential of Heat diffusion for Affinity-based Transition Embedding) [4]; (b) *clustering* and related graph-based methods, such as SPADE (Spanning-tree Progression Analysis of Density-normalized Events) [5], FlowSOM [6], PhenoGraph [7], and X-shift [8]; and (c) inter-population similarity measures based on the *optimal transport* (OT) framework [9–11]. Although dimensionality reduction and clustering methods are well established, they can oversimplify the data, create mathematically well-defined but biologically uninformative groupings, produce visualizations in which distances between populations lack straightforward biological or statistical interpretation, and, in some cases, be computationally highly demanding (see Fig 1).

Previous studies have demonstrated the utility of high-dimensional FC combined with dimensionality reduction techniques to identify distinct immunotypes in patients [12]. In their work, Mathew et al. analyzed 200 immune features across healthy individuals, recovered and acutely ill COVID-19 patients, revealing three immunotypes with varying degrees of T- and B-cell activation that correlated with disease severity and clinical outcomes. These insights were primarily driven by visual exploration of immune marker distributions in UMAP and t-SNE space, highlighting the power of visualization in immunophenotyping. However, such projection-based methods can be limited in their ability to compare immune states across individuals or over time in a quantitative manner.

Despite its power to compare high-dimensional marker distributions, optimal transport (OT) has seen relatively limited use in flow cytometry analysis. Orlova et al. were among the first to apply Earth Mover’s Distance (EMD) to quantitatively compare biomarker-expression distributions across cell populations, revealing clinically relevant shifts [11]. The optimalFlow framework extended this approach by clustering cytometry data and computing Wasserstein barycenters to generate prototype templates, thereby improving supervised cell-population identification despite biological and technical variability [13]. More recently, CytOpT used a regularized Wasserstein metric to estimate cell-population proportions across samples while accounting for technical variation [14]. In related work, Mukherjee et al. applied persistent homology to flow cytometry data from COVID-19 patients and healthy donors, using selected marker combinations to construct persistence diagrams that capture topological features such as connected components and cycles [15]. Although this framework enables rigorous sample comparison via Wasserstein distances, it is not a direct OT-based analysis of cell-distribution geometry, and the resulting persistent-homology representations may not be easily interpretable in biological terms or visually intuitive for clinical use. Taken together, these studies highlight the promise of OT and related Wasserstein-based comparisons for cytometry analysis, while also underscoring the need for broader adoption and improved interpretability in the field.

Although OT techniques have seen limited use in analytical flow cytometry, their application to direct data visualization remains even rarer. Notable examples include CytoMDS, which combines the EMD with classical MDS to generate low-dimensional representations for visual quality control [9], and the framework proposed by Gachon et al. [10], which integrates Wasserstein PCA with log-ratio PCA to produce biologically informed embeddings of high-parameter FC data, thereby supporting minimal residual disease detection and clustering in leukemia cohorts.

In this report, we introduce visualization techniques for multiparameter FC data that are robust, mathematically rigorous, biologically interpretable, and amenable to quantitative evaluation. These methods are essential for communicating biological findings, as they transform complex datasets into clear insights and reveal meaningful patterns and relationships. Effective visualizations also enhance communication and collaboration among researchers and clinicians by providing a shared framework for interpreting results.

Our approach uses an entropically regularized OT measure, commonly known as the Sinkhorn distance [16]. The central idea is that each cell population’s phenotype is represented by its marker-expression profile. Populations with similar marker-expression distributions can be aligned at lower cost, leading to smaller OT values, whereas more dissimilar populations incur higher transport costs. In this framework, transport costs are defined directly in marker-expression space. Because classical OT can be prohibitively expensive for large, high-dimensional datasets, entropic regularization is used to smooth the optimization problem and enable efficient computation via the Sinkhorn–Knopp algorithm, which provides a fast approximation to the unregularized OT solution.

We encode pairwise Sinkhorn distances between cell populations in a graph-based framework that consolidates information otherwise dispersed across multiple two-dimensional dot plots and other conventional displays into a single representation. This representation is intended to be informative for researchers while remaining accessible to non-specialist users, including clinicians. It also enables both visual and quantitative comparison of FC samples through graph edit distance (GED). Unlike manifold-learning approaches such as t-SNE and UMAP, which are primarily used to explore cellular heterogeneity, our framework is not intended to discover novel cell populations. Instead, it supports quantitative, comparative, and longitudinal analysis of predefined phenotypes, where interpretability and statistical rigor are essential. At the core of the method is an efficiently computable OT measure for quantifying dissimilarity between cell populations, making the resulting graph representation well suited to monitoring disease progression and treatment effects.

## 2. Materials and methods

This section first details the flow cytometry datasets used to showcase our visual and quantitative analyses, then presents a clear, step-by-step description of the computational workflow.

### 2.1. Data acquisition and preprocessing

#### 2.1.1. Malignant peritoneal mesothelioma dataset.

Flow cytometry data illustrating malignant peritoneal mesothelioma (MPM) is derived from a clinical trial of adjuvant dendritic cell–based immunotherapy (DCBI) conducted at Erasmus MC Cancer Institute in Rotterdam, Netherlands [17]. Fourteen patients were vaccinated at three time points, each two weeks apart, beginning 8–10 weeks after their cytoreductive surgery with hyperthermic intraperitoneal chemotherapy (CRS-HIPEC). Peripheral blood mononuclear cells (PBMCs) were collected from two cohorts (Group 1, N = 9; Group 2, N = 5) at baseline (pre-vaccination), two weeks post-first vaccine, and two weeks post-third vaccine. All samples were analyzed across six 14-color flow-cytometry panels and stored as FCS files.

Raw FCS files were processed in the R (v4.4.1) environment following the preprocessing pipeline of Dietz et al. [17]. Briefly, we removed margin events and filtered the data using PeacoQC [18], applied fluorescence signal unmixing (compensation), and rescaled marker abundances. Manual gating of Lymphocyte subpopulations was performed in FlowJo v10.10.0 [19]. Finally, data were harmonized using CytoNorm [20].

#### 2.1.2. Acute myeloid leukemia dataset.

The Acute Myeloid Leukemia (AML) dataset originates from the FlowCAP-II initiative (Flow Cytometry: Critical Assessment of Population Identification Methods), which was established to benchmark and evaluate computational pipelines for distinguishing AML from healthy samples [21]. This cohort comprises flow cytometry data from 359 individuals (316 healthy controls and 43 AML patients), acquired across eight 7-color panels and archived as FCS files. The raw data, already compensated and transformed by the repository, were further processed by scaling marker intensities and removing margin events before downstream analysis. The complete dataset is publicly accessible via FlowRepository: http://flowrepository.org/id/FR-FCM-ZZYA.

### 2.2. Cell population identification

Manual gating is the most traditional technique for identifying and quantifying specific cell populations in flow cytometry. It involves visually inspecting compensated fluorescence dot plots and delineating “gates” around populations of interest. Although intuitive and highly flexible, manual gating is labor-intensive and inherently subjective, which limits its scalability for high-throughput studies. Automated gating algorithms (including machine learning approaches) expand this framework and offer greater efficiency and reproducibility across large, complex datasets [22].

To manage the burden of manual annotation, researchers often perform gating or clustering in low-dimensional embeddings produced by manifold learning methods, such as t-SNE and UMAP. However, these projections can distort true inter-population relationships, reducing interpretability.

Unsupervised clustering techniques (such as DBSCAN [23], FlowSOM [6], FlowGrid [24], and model-based approaches like LAMBDA [25]) provide another alternative to manual gates by grouping cells based on density or probabilistic models. Despite all these advances, many biologists still prefer manual gating for its direct control over population boundaries and its transparent, easily explained results.

Crucially, our visualization framework is agnostic to the upstream method of cell population assignment: it requires only that each cell be labeled, whether through manual gating, clustering, or other classifiers. While our examples assume discrete cell type labels, the approach naturally extends to probabilistic identities, in which each cell is represented by a vector of class-membership probabilities. We do not explore this extension here, as it falls beyond the current scope.

**Fig 2:**
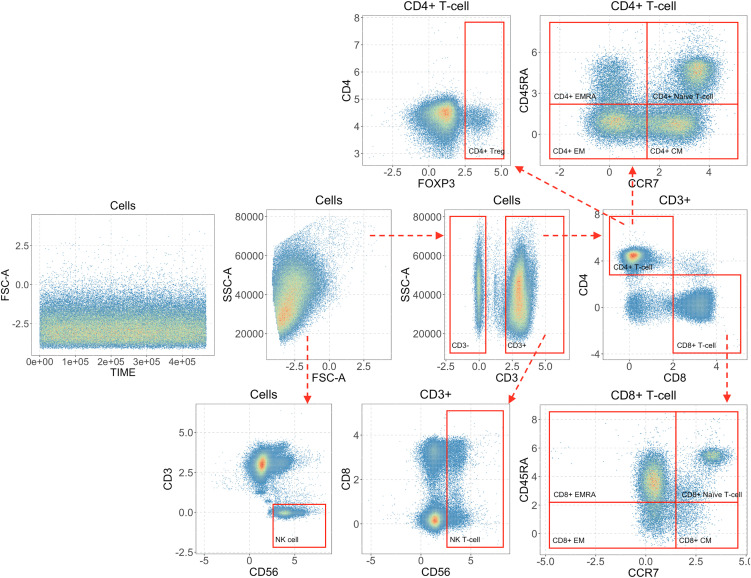
An illustration of the semi-automated gating process that identifies natural killer (NK) cell and T-cell populations in an MPM patient post-first vaccine (Patient ID: MCV005).

Our preprocessing employs a semi-automated, knowledge-driven pipeline for cell type assignment using a template of manually established gates. Beginning with a batch of normalized FCS samples and their associated cytometry panel, we first enumerate the candidate cell populations informed by marker panels and published phenotype definitions. From these descriptions, we compile a gating schema that specifies the marker–expression status of each population (e.g., positive vs. negative, high vs. low). We then apply this schema to one or a few representative samples, defining precise fluorescence-intensity thresholds (rectangular gate boundaries; see Fig 2). Because all samples are harmonized before processing, these thresholds transfer directly across the dataset.

Next, we encode the gating thresholds as conditional statements in our processing workflow, automatically assigning each cell to a defined population based on its measured marker intensities. This approach is panel-specific, tunable, and transparent. It preserves expert knowledge rather than inferring clusters purely from data, yet it is far more efficient than fully manual gating. Importantly, as mentioned earlier, our downstream quantification and visualization steps are agnostic to the choice of cell-assignment method, provided that every cell carries a population label. Finally, these explicit phenotype definitions form the backbone of our visualization framework, as described in the following section.

### 2.3. Inter-population Sinkhorn distance computation

After identifying the cell populations in a cytometry sample, we calculate the Sinkhorn distances between each pair of populations. Full mathematical details on the optimal transport framework and the Sinkhorn distance computation are provided in the S1 Text. Here, we offer a concise overview of the process.

In brief, consider two cell populations, *C*_1_ and *C*_2_, containing *n*_1_ and *n*_2_ cells, respectively. We assign each cell in *C*_1_ a mass of 1/*n*_1_ and each cell in *C*_2_ a mass of 1/*n*_2_, so that both distributions sum to one. Next, we construct the cost matrix


$$\begin{array}{c} M\in {\mathbb{R}}^{n_{1}\times n_{2}},\;M_{ij}=\|x_{i}^{\left(1\right)}-x_{j}^{\left(2\right)}{\|}_{2}, \end{array}$$

where $x_{i}^{\left(1\right)}$ and $x_{j}^{\left(2\right)}$ are the marker-expression vectors of cells *i* and *j* in *C*_1_ and *C*_2_, respectively.

We then solve the entropically regularized optimal transport problem (see the S1 Text) using the Sinkhorn-Knopp algorithm to obtain the transport plan $P^{\lambda }\in {\mathbb{R}}^{n_{1}\times n_{2}}$. The Sinkhorn distance is given by


$$\begin{array}{c} D_{M}^{\lambda }(C_{1},C_{2})\;=\;\sum_{i=1}^{n_{1}}\sum_{j=1}^{n_{2}}P_{ij}^{\lambda }\;M_{ij}, \end{array}$$

which quantifies the minimum “work” required to morph the distribution of *C*_1_ into that of *C*_2_. Note that $D_{M}^{\lambda }$ is a dual-Sinkhorn divergence, but to keep the terminology consistent with the original Sinkhorn distance paper [16], we refer to it as Sinkhorn distance in our work.

Here, λ is the entropic regularizer parameter. Smaller values of λ yield Sinkhorn distances that more closely approximate the Wasserstein distance, at the expense of increased computational cost and potential numerical instability. To assess this, we evaluated $D_{M}^{\lambda }$ for λ={0.01,0.05,0.1,0.5,1.0,2.0,5.0} and selected λ=0.1 as a trade-off point that provides numerical stability while preserving biologically meaningful structure. By normalizing each population’s total mass to one and leveraging entropic regularization for rapid convergence, Sinkhorn distance captures rich, high-dimensional differences in marker abundances, providing a rigorous and interpretable foundation for our visualization and comparison workflows.

### 2.4. Graph-based data visualization

Once we obtain the Sinkhorn distances between the cell populations, we visualize the cytometry samples using graphs. In this report, we adopt the standard mathematical definition of a graph *G* = (*V*, *E*), where *V* is the set of vertices (also called nodes) and *E* is the set of edges linking pairs of vertices. For example, in a social network graph, each node represents an individual, and each edge represents a friendship, enabling the analysis of connectivity, community structure, or influence. By the same convention, in our flow cytometry framework, each vertex corresponds to a distinct cell population.

Crucially, each edge in our graphs carries two attributes:

1. **Weight:** Visually encoded as edge thickness (and color), proportional to the inter-population Sinkhorn distance ${𝒲}_{\varepsilon }(C_{i},C_{j})$, which quantifies distributional differences in marker expression between cell populations $C_{i}$ and $C_{j}$.
2. **Length:** Determined by the phenotype distance $d_{H}(\phi_{i},\phi_{j})$, computed via the Hamming distance [26] between expert-defined phenotype strings ($\phi_{i}$ and $\phi_{j}$, for cell populations $C_{i}$ and $C_{j}$, respectively), so that populations with more similar marker-expression patterns lie closer together. The process of computing $d_{H}(\phi_{i},\phi_{j})$ is described below.

This dual encoding ensures that both the cost of “transporting” one distribution into another and the categorical phenotypic similarity are represented simultaneously and unambiguously.

For graph-based visualization, we leverage R’s *ggplot2* and *igraph* packages [27,28]. The *igraph* library supplies a high-performance suite for constructing, manipulating, and analyzing graphs, complete with numerous layout algorithms and network statistics. However, its built-in layout algorithms often rely on random initialization, leading to inconsistent representation across runs, which is problematic for comparative cytometry visualizations. To ensure both reproducibility and biological relevance, we instead compute domain knowledge-informed layouts, using Hamming distances to deterministically position vertices in a manner that reflects underlying cell phenotype relationships as understood by immunologists. By decoupling layout (Hamming) from edge-weight encoding (Sinkhorn), our approach guarantees both reproducibility and a clear, interpretable representation of phenotypic relationships.

#### 2.4.1. Phenotype-aware layout.

Central to our visualization framework is the explicit encoding of expert-defined phenotype descriptors as fixed-length strings, one entry per marker in the cytometry panel. For example, suppose our panel comprises the markers


$$\begin{array}{c} \{CD3,\;CD19,\;CD27,\;CD38,\;IgD,\;IgM,\;CD5,\;CD24\}. \end{array}$$

We represent the regulatory B-cell phenotype


$$\begin{array}{c} {CD3}^{-},\;{CD19}^{+},\;{CD27}^{+},\;{CD38}^{high},\;{IgD}^{-},\;IgM\;(DC),\;{CD5}^{+},\;{CD24}^{+} \end{array}$$

by the string [−,+,+,++,−,DC,+,+].

Here, “DC” (for “Don’t Care”) originates from Boolean algebra and digital-circuit design, where it denotes a variable whose value (0 or 1) does not affect the logical outcome. In our phenotype strings, “DC” indicates that the corresponding marker (IgM) is irrelevant for defining this population.

We then compute the *phenotype distance* between any two populations via the Hamming distance, but excluding positions labeled “DC” in either string. For instance, the strings


$$\begin{array}{c} {\text{IgM}}^{+}\text{ memory B-cell: }[-,+,+,-,-,+,DC,DC] \end{array}$$

and


$$\begin{array}{c} \text{Class-switched memory B-cell: }[-,+,+,-,-,-,DC,DC] \end{array}$$

differ only at the IgM position (between “+” and “–”), yielding a phenotype distance of $d_{H}=1$. Note that the classical Hamming distance counts the number of differing positions between two strings. However, when computing phenotype distances between cell populations, we can customize these distances to reflect biological relevance.

Suppose we denote marker expression levels as follows: “++” indicates high expression, “+” standard expression, “low” low expression, and “-” no expression. We then assign a uniform distance of 1 between consecutive expression levels. Accordingly, the distance between “++” and “+” is 1, whereas the distance between “++” and “-” is 3. Users have the flexibility to define both the marker expression levels and the distances between consecutive expression levels (see Section 2.6). Don’t Care (DC) states are used when a marker is present in the flow cytometry panel but is not required to define a particular cell population. Hamming distances are computed over the full set of panel markers, with DC states contributing neither matches nor mismatches. This ensures a consistent and comparable distance scale across all population pairs.

These pairwise phenotype distances form a symmetric matrix, denoted as *H*. To produce a reproducible, biologically informed two-dimensional layout, we apply the Kamada–Kawai algorithm [29] to *H*. The Kamada-Kawai layout algorithm computes a force-directed layout by solving a nonlinear system of equations iteratively, yielding a fixed configuration of vertices (one per cell population) such that Euclidean separations approximate the phenotype distances. By anchoring each population at these deterministic coordinates, we eliminate layout variability and ensure that vertex positions carry direct interpretive meaning across all samples.

With vertex positions fixed, each cytometry sample is visualized as a weighted graph: vertex radii scale with the population’s fraction of total cells, and edges encode the inter-population Sinkhorn distances (See S1 Text) through thickness and color. This single graph representation unifies high-dimensional marker-expression profiles and population frequencies into a compact, interpretable summary, eliminating the need for multiple two-dimensional dot plots while preserving both biological and statistical insights.

### 2.5. Inter-sample GED computation

Up to this point, we have focused on methods for representing and visualizing individual flow cytometry samples. However, assessing the similarity or dissimilarity between pairs of samples can yield valuable insights, such as distinguishing healthy from diseased states, understanding disease mechanisms and progression, and evaluating treatment effects. Our graph-based representation enables inter-sample comparisons in a computationally efficient and biologically meaningful manner by leveraging the concept of graph edit distance (GED). GED quantifies the minimum cost required to transform one graph into another through a sequence of edit operations, such as adding, deleting, or substituting vertices and edges. As a well-established tool in pattern recognition, machine learning, and network analysis, GED provides a robust framework for comparing graph structures. However, because exact GED computation is NP-hard, we adapt the approximate GED implementation from Python’s *NetworkX* library [30], based on the method proposed by [31], which applies depth-first search (DFS) with pruning over the space of partial edit paths.

In our approach, we enhance the biological relevance of GED by assigning custom costs to edit operations. Vertex edit costs ($c_{V}$) are designed to reflect changes in cell-population proportions (p(·)). Specifically, vertex insertion and deletion incur costs equal to the corresponding population proportion, whereas vertex substitution incurs a cost equal to the absolute difference between the population proportions in the two samples. All vertex edit costs are scaled by a weight factor $w_{V}$:


$$\begin{array}{c} \text{Vertex insertion: }c_{V}(\emptyset \to v_{j})=w_{V}\;p(v_{j}),\;\text{Vertex deletion: }c_{V}(v_{i}\to \emptyset )=w_{V}\;p(v_{i}),\;\text{Vertex substitution: }c_{V}(v_{i}\to v_{j})=w_{V}\;|p(v_{i})-p(v_{j})|. \end{array}$$ (1)

Edge edit costs ($c_{E}$) are defined analogously to capture changes in inter-population phenotypic relationships, represented here by Sinkhorn distances s(·). Edge insertion and deletion incur costs equal to the corresponding Sinkhorn distance, whereas edge substitution incurs a cost equal to the absolute difference between Sinkhorn distances in the two samples. All edge edit costs are scaled by a weight factor $w_{E}$:


$$\begin{array}{c} \text{Edge insertion: }c_{E}(\emptyset \to e_{ij})=w_{E}\;s(e_{ij}),\;\text{Edge deletion: }c_{E}(e_{ij}\to \emptyset )=w_{E}\;s(e_{ij}),\;\text{Edge substitution: }c_{E}(e_{ij}\to e_{kl})=w_{E}\;|s(e_{ij})-s(e_{kl})|. \end{array}$$ (2)

The total GED between two sample graphs *G*_1_ and *G*_2_ is then defined as the minimum total cost over all valid edit paths transforming *G*_1_ into *G*_2_:


$$\begin{array}{c} GED(G_{1},G_{2})=\min_{\gamma \in \Gamma (G_{1},G_{2})}\left(\sum_{o\in \gamma_{V}}c_{V}(o)+\sum_{o\in \gamma_{E}}c_{E}(o)\right), \end{array}$$ (3)

where $\Gamma (G_{1},G_{2})$ denotes the set of all valid edit paths between *G*_1_ and *G*_2_, and $\gamma_{V}$ and $\gamma_{E}$ denote the vertex and edge edit operations in a given path, respectively.

Assigning equal weighting factors to vertex and edge edit costs ($w_{V}=w_{E}$) assigns the same nominal importance to changes in population proportions and changes in inter-population phenotypic relationships. In the absence of a priori biological knowledge favoring one source of variation over the other, this choice provides a simple and interpretable default. However, the GED framework readily accommodates alternative weighting schemes when domain-specific knowledge suggests differential importance of vertex- or edge-level changes.

For all experiments presented in this work, population proportions are scaled to lie in the interval [0,1], and inter-population Sinkhorn distances are *L*_1_-normalized to emphasize relative phenotypic differences across population pairs. The vertex and edge edit cost weight factors ($w_{V}$ and $w_{E}$) are both set to 1. Under this normalization, vertex and edge edit operations may still contribute on different numerical scales; therefore, equal weighting should be interpreted as equal modeling emphasis rather than equal absolute contribution. Users may adopt alternative normalization strategies and weighting choices as appropriate for their data and experimental objectives.

In the datasets considered in this study, prior knowledge of cell populations ensured that no populations emerged or vanished across samples. Consequently, the GED computation reduces to summing vertex and edge substitution costs, yielding a linear-time procedure with negligible runtime (on the order of milliseconds per sample pair). The approximate GED algorithm implemented in *NetworkX* was evaluated only for validation purposes and required several minutes per comparison for graphs of comparable size.

The above sections describe the core computational components of our framework, including cell population identification, inter-population distance computation, graph-based visualization, and inter-sample comparison. We next summarize the computational prerequisites required to apply this method in practice.

### 2.6. Computational prerequisites

**Fig 3:**
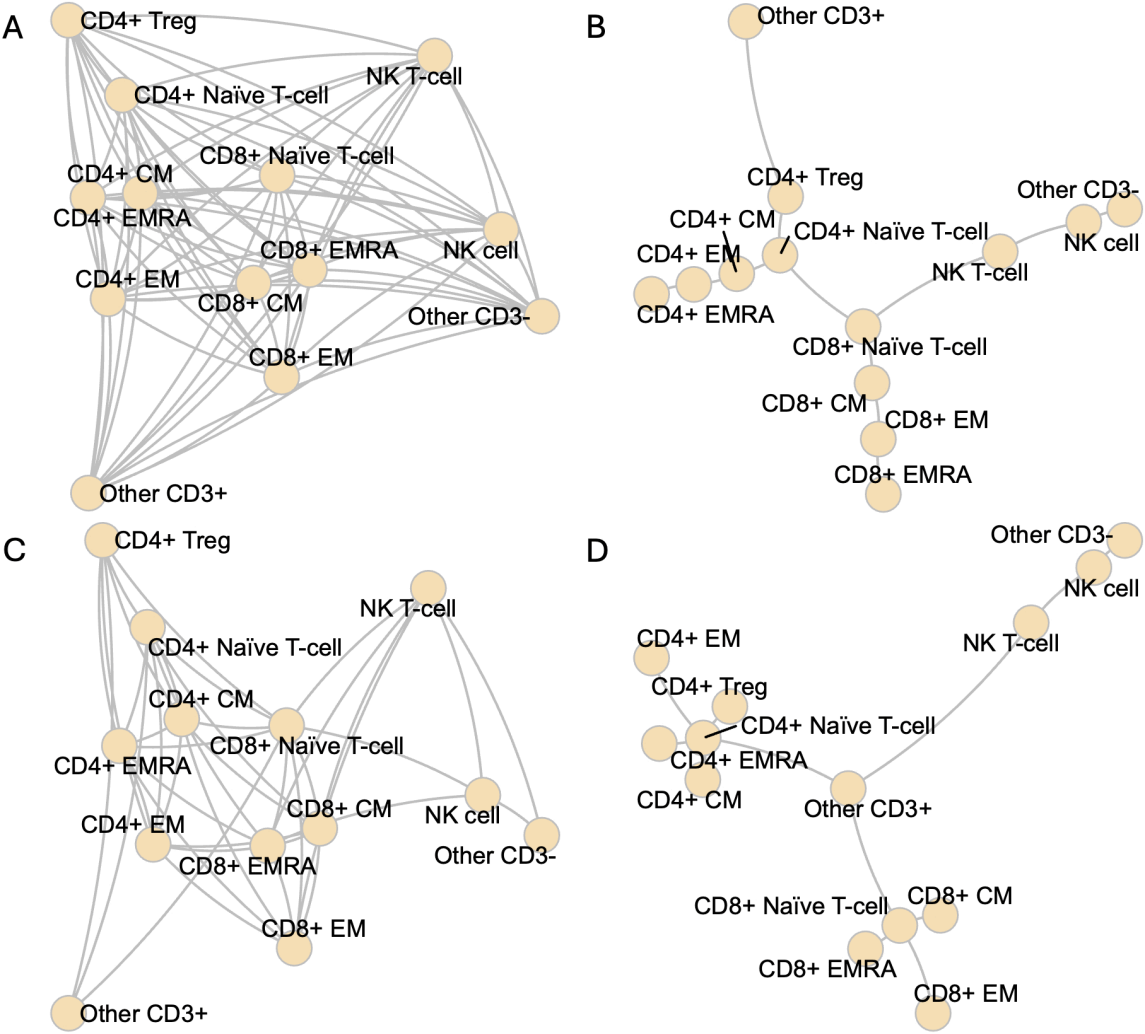
A collection of phenotype-aware layouts for the graph-based visualization of the NK cell and T-cell populations. **(A)** Fully-connected layout. **(B)** Minimum spanning tree layout. **(C)** Distance-threshold layout. **(D)** Expert-curated layout.

The implementation of the proposed framework assumes access to preprocessed high-dimensional flow cytometry data and relies on the following components:

- **Single-cell data and population assignments:** Each cell must be represented as a marker-expression vector, with standard preprocessing steps (compensation, transformation, and quality control) completed before analysis. Cells must also be assigned to populations through manual gating, semi-automated gating, or unsupervised clustering.
- **Cytometry panel and phenotype definitions:** The cytometry panel defining the set of markers must be known for each dataset. For every identified population, a phenotype specification describing marker-expression states (e.g., ++, + , low, -) is required. These phenotype definitions are stored in a tabular format, for example, an Excel file (rows: populations; columns: markers), and are used to compute phenotype-aware Hamming distances that determine vertex placement in the graph layout.
- **Graph topology specification:** Graph construction requires the definition of connectivity between cell populations. By default, the framework supports fully connected graphs, minimum spanning trees, and distance-threshold graphs (see Fig 3). For expert-curated or customized visualizations, an explicit adjacency matrix defining the desired topology should be provided in tabular form, for example, as an Excel file.

Together, these inputs enable reproducible computation of inter-population distances, construction of sample-level graphs, and downstream visualization and graph edit distance-based comparisons.

The following sections present the resulting visual summaries and quantitative measures using the MPM and the AML dataset (described in Section 2.1).

## 3. Results

### 3.1. Optimal transport-based visualization for MPM dataset

To illustrate our computational framework, we analyzed a subset of the MPM dataset using three cytometry panels optimized for T-cell profiling: *Co-inhibition*, *Co-stimulation*, and *Cytokine* panel.

- **Core cell-type markers (all panels):** CD56, CD3, CD4, FoxP3, CD8, CD45RA, CCR7, and Live/Dead viability channel.
- **Panel-specific cell-state markers:**
  - *Co-inhibition:* LAG3, PD-1, TIM-3, CD39, KI-67, CTLA-4
  - *Co-stimulation:* CD28, CD137, PD-1, HLA-DR, ICOS, KI-67
  - *Cytokine:* PD-1, TBET, IL-10, TNF-α, IL-2, IFN-γ

Using the shared core markers, our semi-automated gating procedure identified thirteen cell populations:

- Natural killer cells (NK)
- Naural killer T cells (NKT)
- CD4^+^ regulatory T-cells (Tregs)
- CD4^+^ effector memory (EM) T-cells
- CD4^+^ terminally differentiated effector memory (EMRA) T-cells
- CD4^+^ central memory (CM) T-cells
- CD4^+^ naïve T-cells
- CD8^+^ EM T-cells
- CD8^+^ EMRA T-cells
- CD8^+^ CM T-cells
- CD8^+^ naïve T-cells
- Uncategorized CD3^+^ cells
- Uncategorized CD3^–^ cells

Please refer to Section 2.2 and Fig 2 for additional details.

Fig 3 illustrates four phenotype-aware two-dimensional layouts derived from the marker-expression profiles of the identified cell populations. To generate these layouts, we first computed the inter-population phenotype distances (Section 2.4) and encoded them as weights on the edges of a fully connected graph. When this graph is laid out using a force-directed algorithm (Fig 3A), populations with smaller distances naturally cluster closer together, but the large number of edges obscures the most informative biological relationships.

To distill the graph into a more interpretable structure, we explored three edge-filtering strategies. First, extracting a minimum spanning tree (Fig 3B) preserves the strongest links required to maintain connectivity while avoiding redundant cycles. Second, applying a distance threshold (Fig 3C) prunes all edges whose weights exceed a chosen cutoff; for illustration, we use the average edge weight, although other meaningful cutoffs could also be used. Third, expert immunological knowledge guides the selection of edges identified as the most biologically meaningful (Fig 3D). For the remainder of this report, we adopt the expert-curated layout (Fig 3D) to ensure that our visualizations emphasize the relationships most relevant to disease monitoring and treatment response.

#### 3.1.1. Graph visualization and comparison of FC samples.

**Fig 4:**
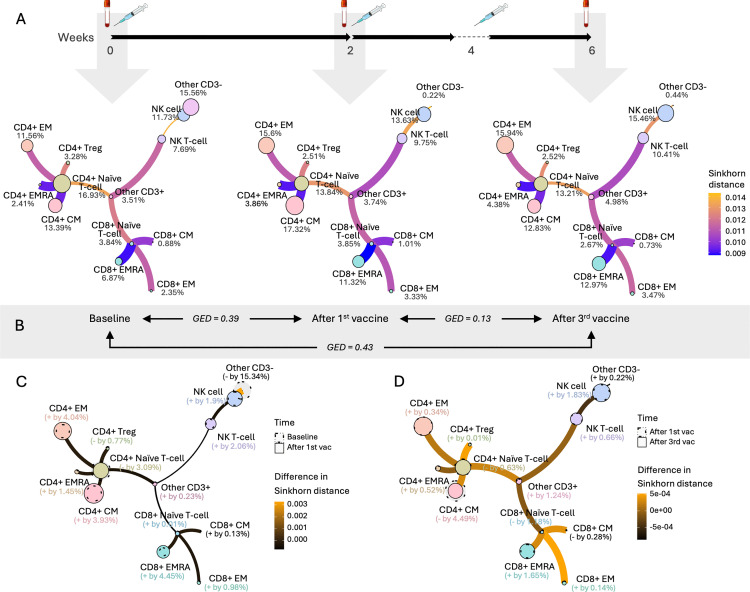
Sinkhorn distance-based graph representations for longitudinal comparison of T-cell populations in patient MCV005 following dendritic cell-based immunotherapy (DCBI). **(A)** Flow-cytometry-derived population graphs constructed from samples collected at baseline (week 0), 2 weeks after the first vaccination (week 2), and 2 weeks after the third vaccination (week 6). The timeline bar indicates the timing of vaccine administration and blood-sample collection [32,33]; downward arrows mark the time points corresponding to the displayed graphs. In each graph, vertices represent T-cell populations, and edges encode inter-population Sinkhorn distances. Edge color and thickness are mapped to Sinkhorn distance using a single shared color scale across all three graphs, enabling direct visual comparison across time points. **(B)** Pairwise GEDs computed between the three graphs shown in panel **A. (C, D)** Comparison graphs visualizing pairwise changes between consecutive time points: baseline versus after the first vaccination (panel **C**) and after the first versus after the third vaccination (panel **D)**. In each comparison graph, vertices from the earlier time point are shown as transparent dotted outlines, whereas vertices from the later time point are shown as solid colored nodes, allowing changes in vertex size to be assessed visually. Edge color encodes differences in inter-population Sinkhorn distances between the corresponding graph pairs in panel **A.** The color bars in panels **C** and **D** represent changes in Sinkhorn distance, rather than absolute distances, and are scaled independently to emphasize the changes specific to each time-point comparison.

The MPM dataset comprises flow-cytometry samples collected from each patient at three therapy milestones: baseline (pre-vaccination), two weeks after the first vaccine, and two weeks after the third vaccine. In Fig 4A, we visualize patient MCV005’s co-inhibition-panel samples at these three time points using our expert-curated graph layout. Here, each vertex represents one of the thirteen gated Lymphocyte populations, with its radius proportional to that population’s fraction of total lymphocytes. Vertex positions are determined by pairwise phenotype distances, so phenotypically similar populations lie closer together.

Edge weights (but not length) encode the inter-population Sinkhorn distances: smaller distances (indicating greater similarity in marker expression) produce thicker, more vividly colored edges, while larger distances yield thinner, paler edges. Both vertex sizes and edge-weight scalings are normalized on a per-patient basis, ensuring that comparisons across the three time points reflect true biological shifts rather than arbitrary scaling differences.

It is worth noting that two populations may appear close together, reflecting phenotypic (functional) similarity or a lineage relationship, yet still differ in marker-abundance profiles, as indicated by a thinner connecting edge. Although Fig 4A displays individual FC samples, the same graph-based approach has the capacity to be applied to averaged data from multiple samples. This yields a compact, informative visualization that combines biological context with statistical rigor.

From the three time-point graphs in Fig 4A, we observe trends consistent with Dietz et al. [17]:

- Natural killer cells, effector memory (EM) T cells, and terminally differentiated effector memory (EMRA) T cells increase in proportion over the course of treatment.
- Naïve T cells decrease in abundance following the third vaccine.
- Central memory (CM) T cells rise after the first vaccine but decline after the third.

We also observe that several edges, for example the edge connecting NK cells and NKT cells, become thicker and more vividly colored over the course of vaccination. This indicates that the inter-population Sinkhorn distance is decreasing, meaning that the two populations are becoming more similar in their marker-expression profiles. Biologically, this suggests that, during vaccination, the marker-expression patterns of NK cells and NKT cells converge, requiring less conceptual “effort” to transport one population into the other. This convergence may reflect the emergence of shared or overlapping functional states over time.

Although the sample graphs share the same layout and use consistently scaled vertices and edges, comparing two graphs side by side can still be challenging, particularly when changes in cell proportions or inter-population Sinkhorn distances are subtle. To address this, we construct a comparison graph that overlays two FC samples using a shared layout and highlights their differences more effectively. Figs 4C and 4D present visual comparisons between the baseline and post-first-vaccination samples, and between the post-first- and post-third-vaccination samples, respectively. In these comparison graphs:

- Vertices from the two samples are distinguished by style: vertices from one sample are shown as dotted circles, whereas those from the other are shown as solid circles. When the proportion of a population increases, the solid circle encloses the dotted one; when it decreases, the dotted circle is larger. Numeric labels at each vertex indicate the percentage change in that cell population.
- Edges encode the change in Sinkhorn distance between the two samples. A positive edge weight denotes increased similarity (i.e., a decrease in Sinkhorn distance), whereas a negative edge weight denotes decreased similarity (i.e., an increase in Sinkhorn distance).

This visualization style provides a succinct summary of changes in cell populations and their relationships between two time points, offering valuable insight into therapeutic effects. Moreover, this approach extends beyond treatment monitoring to comparisons between healthy and diseased samples or to tracking disease progression, in which specific cell populations may emerge or disappear entirely. Our graph-based methodology readily accommodates such dynamic processes.

Beyond visual comparison, we also incorporate a quantitative measure of similarity between FC samples, namely graph edit distance, as detailed in Section 2.5. Fig 4B presents the GED values for the sample graphs shown in Fig 4A. GED captures changes in both cell proportions and inter-population Sinkhorn distances. While a single GED value may be of limited interpretive value, analysis of GEDs across all three time points and patients within the three T-cell panels reveals more informative patterns, which are explored further in Section 3.1.2.

Note that although these graphs may visually resemble those generated by SPADE, the two approaches differ fundamentally in both methodology and purpose. SPADE clusters cells and constructs a minimum spanning tree to infer cellular hierarchies, relying on density normalization and unsupervised clustering. In contrast, our graph-based visualization uses predefined cell populations and constructs phenotype-aware graphs in which edges are weighted by Sinkhorn distances to reflect distributional differences in marker expression. It also incorporates graph edit distance for quantitative sample comparison, enabling robust tracking of biological changes; these capabilities are not natively provided by SPADE.

#### 3.1.2. Relevance of graph-based visualization and graph edit distances for MPM dataset.

In this section, we align our visualization and quantification framework with the key clinical observations of Dietz et al. [17]. Their trial evaluated adjuvant dendritic cell–based immunotherapy in 14 MPM patients following CRS–HIPEC, using six 14-color flow-cytometry panels sampled at baseline, two weeks post-first vaccine, and two weeks post-third vaccine. Although total lymphocyte and CD8^+^ T-cell frequencies remained stable, DCBI induced a robust memory T-cell response:

- **Effector memory (EM) and central memory (CM) T cells** expanded significantly after the first dose and remained elevated through the third.
- **Naïve T cells** declined sharply following the third vaccine.
- **CD8^+^ EMRA T cells** increased most notably in patients with prolonged progression-free survival.

Dietz et al. summarized these dynamics with grouped boxplots and stacked bar charts (see their Fig 2). While effective for trend analysis, these plots require multiple panels to cover all subpopulations and do not convey the phenotypic relationships among them.

Our graph-based representation (Fig 4) consolidates each sample into a single network:

- **Vertex size:** scales with each subpopulation’s proportion.
- **Vertex position:** determined by phenotype similarity (Hamming distance), so similar populations lie closer together.
- **Edge thickness and color:** encode Sinkhorn distances, capturing distributional shifts in marker expression.

This unified visualization not only reproduces the reported expansion of EM and CM subsets, the decline in naïve cells, and the enrichment of EMRA cells, but also shows how these populations are related phenotypically, thereby enhancing both biological interpretability and statistical rigor in high-parameter cytometry.

Importantly, cell subpopulations in a flow-cytometry sample are not isolated but biologically interconnected. Simply tracking changes in individual cell proportions does not fully capture the complexity of the immune response. By also incorporating changes in Sinkhorn distances between subpopulations, our graph-based method reflects changes in relationships among populations. This provides a more complete view of immune modulation within and between samples.

**Fig 5:**
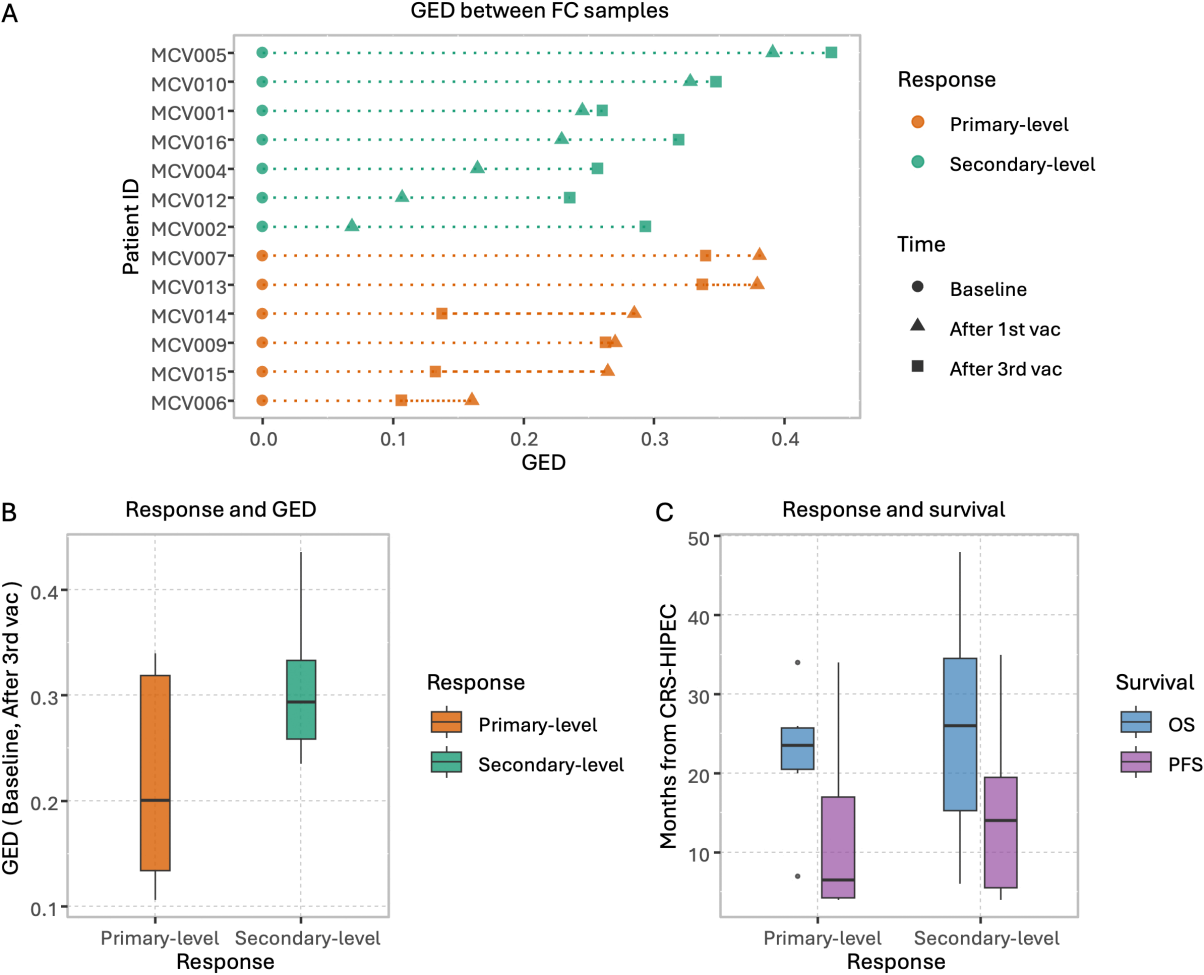
Graph edit distance (GED) insights and clinical outcomes in MPM patients. **(A)** GED trajectories across three vaccination time points for two distinct patient response groups. **(B)** Comparison of overall GED from baseline to the third vaccine between primary-level and secondary-level responders. **(C)** Comparison of progression-free survival (PFS) and overall survival (OS) between primary-level and secondary-level responders.

We calculated GED values for each MPM patient at baseline, 2 weeks after the first vaccination, and 2 weeks after the third vaccination, revealing a heterogeneous pattern across patients (Fig 5A). In some patients, GED increased steadily from baseline to the third vaccination, whereas in others it decreased after an initial increase following the first dose. Because GED quantifies shifts in cell-population structure, these trajectories indicate heterogeneity in immunological change across patients.

Based on these observed GED trajectories, we grouped patients descriptively into those whose GED declined after the third dose (six patients) and those whose GED continued to increase (seven patients) (one patient out of the 14 patients did not complete the clinical trial). This grouping was not pre-specified in the clinical trial and is not intended as a clinical classification, but rather as a descriptive summary of patterns revealed by the GED-based analysis. Fig 5B shows that the group with increasing GED trajectories exhibited a larger overall GED change from baseline to the third vaccination than the group with declining trajectories. We then compared these GED-based patterns with the clinical outcomes reported by Dietz et al. [17], specifically progression-free survival (PFS) and overall survival (OS) after CRS-HIPEC, solely to illustrate how immune-structure dynamics captured by GED may align with known clinical observations. In this descriptive comparison, patients with increasing GED trajectories showed longer PFS than those with declining trajectories (Fig 5C). We emphasize that this comparison is illustrative and does not constitute clinical inference or validation based on GED trends alone.

Because of substantial baseline heterogeneity and the small cohort size, formal statistical inference regarding immunological shifts induced by the immunotherapy regimen is limited. Therefore, for exploratory and hypothesis-generating purposes, we normalized GED trajectories to baseline across patients (Fig 5A) and performed a pseudo-F test on the change in GED from baseline to the third vaccination, which yielded p ≤ 0.001. However, given the limited number of meaningful permutations, this result should be interpreted cautiously as suggestive of immunological shifts rather than as definitive evidence of clinical significance. Importantly, this case study is intended to demonstrate the potential of the proposed framework. In future applications involving larger cohorts and prospective study designs, GED-based trajectory analysis could be incorporated from the outset, enabling more rigorous hypothesis testing and clinically meaningful validation.

A sensitivity analysis assessing the robustness of GED trajectories to variation in the vertex and edge edit-cost weights ($w_{V}$ and $w_{E}$, defined in Section 2.5) is provided in the S1 Text.

### 3.2. Optimal transport-based visualization for AML dataset

**Fig 6:**
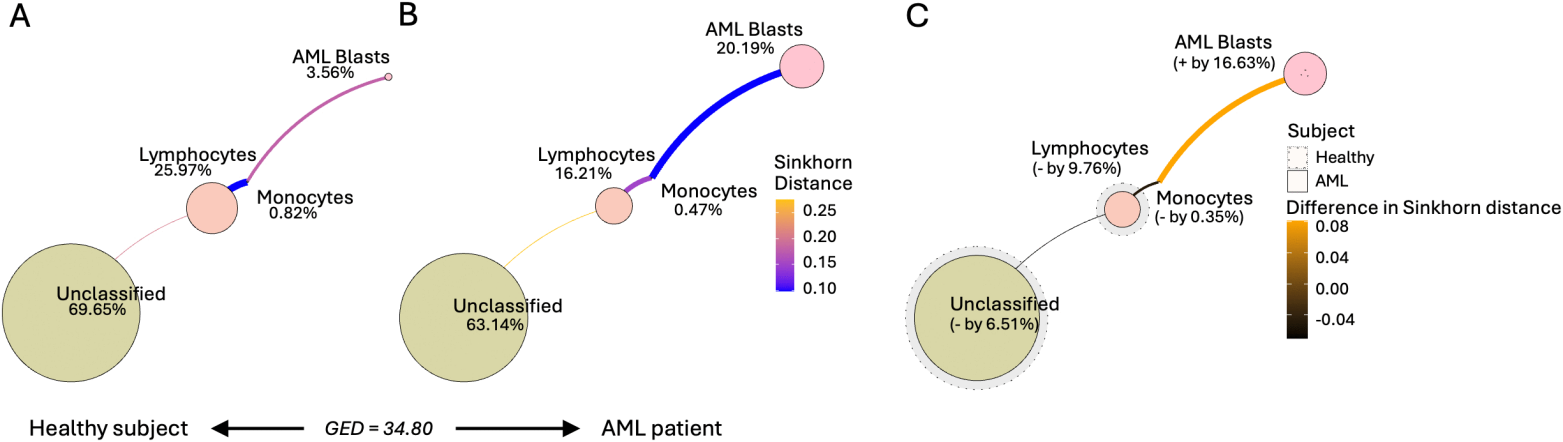
Sinkhorn distance-based visual summary of changes in cell populations in AML dataset using an MST-layout. **(A)** Illustration of an FC sample graph of a healthy subject (Individual 1). **(B)** Illustration of an FC sample graph of an AML patient (Individual 5). **(C)** Visual comparison of sample graphs between healthy (Fig 6A) and diseased (Fig 6B). The graph edit distance (GED) between the graphs in Fig 6A and 6B is shown.

To demonstrate the versatility of our framework beyond treatment monitoring in the MPM dataset, we applied it to flow cytometry data from a publicly available acute myeloid leukemia (AML) dataset to distinguish healthy and diseased profiles. This AML dataset comprises eight cytometry panels, each measuring five markers; here, we focus on panel 6 (HLA-DR, CD117, CD45, CD34, CD38) to identify AML blasts (myeloblasts), monocytes, and lymphocytes. Notably, the lymphocyte and monocyte gates were confirmed using markers from panel 7. Given the smaller number of populations relative to the MPM study, we constructed a phenotype-aware minimum spanning tree (MST) for the graph-based visualization. Fig 6 presents MST-based graphs for a healthy donor (Individual 1), an AML patient (Individual 5), and their direct comparison. In the AML patient, lymphocyte proportions are markedly reduced, whereas the myeloblast population is prominently expanded. These findings are consistent with the characteristic immunophenotypic features of AML.

## 4. Discussion

In the work described in this report, our primary objective was to develop an interpretable and quantifiable visualization technique for high-parameter flow cytometry. We deliberately de-emphasized the cell-classification step, working under the assumption that our visualizations would be applied downstream of diverse pre-processing pipelines. Accordingly, we used a straightforward semi-automated gating procedure to assign cell populations, ensuring consistency with strategies previously applied to these data. This design choice reflects the fact that our framework is intended as a downstream analytical and visualization framework for comparing established cell populations, rather than as a method comparable to unsupervised manifold-learning approaches for discovering new cell types. Although manifold-learning approaches such as t-SNE and UMAP are unsupervised, their biological interpretation in practice often relies on mapping identified clusters back to predefined cell populations through gating or marker-based annotation. Our method incorporates this prior knowledge directly into the analytical framework, enabling interpretable and biologically grounded comparisons.

While the implementation of advanced classification or clustering methods lies beyond the scope of this work, we acknowledge that integrating our phenotype-aware layouts and inter-population Sinkhorn distances into existing frameworks (e.g., FlowSOM) could further enhance the interoperability and analytical robustness of flow-cytometry workflows. FlowSOM provides an unsupervised framework for identifying cell populations, and its graph visualization is derived from the topology of the self-organizing map (SOM) and its minimum spanning tree representation. As a result, vertex placement reflects SOM neighborhood structure rather than explicit biological dissimilarities between populations. In contrast, our proposed phenotype-aware layout embeds populations in a metric space defined by Hamming distances over population phenotype descriptions, thereby encoding interpretable inter-population relationships directly into the graph geometry. These two approaches are therefore complementary: integrating phenotype-aware layouts and inter-population Sinkhorn distances into FlowSOM-derived graphs could preserve the clustering strengths of FlowSOM while enhancing biological interpretability and cross-sample comparability.

From a computational standpoint, we employ the entropically regularized Sinkhorn distance to quantify similarity (or dissimilarity) between cell subpopulations within each flow-cytometry sample. These pairwise distances are then encoded as edge weights in a graph, enabling comparison of entire samples through graph edit distance. Although OT can, in principle, be applied directly to whole samples without introducing a graph abstraction, such an approach presents several important challenges. First, solving a single OT problem between high-parameter samples can be computationally and memory intensive. Second, incorporating cell-population and phenotypic information into the transport cost requires a careful problem-specific formulation. Third, when specific functional populations are not explicitly identified, the resulting OT solution is more difficult to interpret biologically, motivating an additional framework for translating global OT differences into population-level changes.

We acknowledge these challenges and plan to explore a structured, end-to-end OT framework for sample-to-sample comparison in future work. In addition, we intend to investigate alternative inter-population similarity measures, such as Marker Enrichment Modeling (MEM) [34], which quantitatively characterizes cell populations by marker enrichment relative to a reference. Integrating MEM-derived enrichment scores into our graph structures may provide a more interpretable and biologically grounded basis for analyzing graph edit distances, thereby improving our understanding of population-level changes across samples.

## 5. Conclusions

We have introduced a novel, biologically grounded framework for visualizing high-parameter flow cytometry data using optimal transport theory. By using the Sinkhorn distance, we quantified inter-population similarities and encoded these relationships in a graph-based visualization. Our approach combines phenotype-aware layouts with Sinkhorn distances to provide compact, quantitatively rigorous summaries of cell-population structure, enabling direct comparisons across samples.

When applied to a dendritic cell-based immunotherapy trial in malignant peritoneal mesothelioma, our framework reproduced previously reported immunological trends, including memory T-cell expansion and naïve T-cell decline, and identified additional potentially informative patterns of patient stratification. In particular, the observed correspondence between GED patterns and progression-free survival suggests that the method may be useful for immune monitoring in longitudinal studies, although this finding remains exploratory and requires validation in larger cohorts.

Overall, this work bridges mathematical rigor and biological interpretability, laying the groundwork for more actionable analyses in clinical cytometry and immunology research.

## Supporting information

S1 Text Optimal transport framework, GED trajectory sensitivity analysis, and details on Computational parameters. (PDF)

## References

[1] Konecny AJ, Mage PL, Tyznik AJ, Prlic M, Mair F. OMIP-102: 50-color phenotyping of the human immune system with in-depth assessment of T cells and dendritic cells. Cytometry A. 2024;105(6):430–6. doi: 10.1002/cyto.a.24841 38634730 PMC11178442

[2] Van der Maaten L, Hinton G. Visualizing data using t-SNE. Journal of Machine Learning Research. 2008;9(11).

[3] McInnes L, Healy J, Saul N, Großberger L. UMAP: Uniform Manifold Approximation and Projection. JOSS. 2018;3(29):861. doi: 10.21105/joss.00861

[4] Moon KR, van Dijk D, Wang Z, Gigante S, Burkhardt DB, Chen WS, et al. Visualizing structure and transitions in high-dimensional biological data. Nat Biotechnol. 2019;37(12):1482–92. doi: 10.1038/s41587-019-0336-3 31796933 PMC7073148

[5] Qiu P, Simonds EF, Bendall SC, Gibbs KD Jr, Bruggner RV, Linderman MD, et al. Extracting a cellular hierarchy from high-dimensional cytometry data with SPADE. Nat Biotechnol. 2011;29(10):886–91. doi: 10.1038/nbt.1991 21964415 PMC3196363

[6] Van Gassen S, Callebaut B, Van Helden MJ, Lambrecht BN, Demeester P, Dhaene T, et al. FlowSOM: Using self-organizing maps for visualization and interpretation of cytometry data. Cytometry A. 2015;87(7):636–45. doi: 10.1002/cyto.a.22625 25573116

[7] Levine JH, Simonds EF, Bendall SC, Davis KL, Amir ED, Tadmor MD, et al. Data-Driven Phenotypic Dissection of AML Reveals Progenitor-like Cells that Correlate with Prognosis. Cell. 2015;162(1):184–97. doi: 10.1016/j.cell.2015.05.047 26095251 PMC4508757

[8] Samusik N, Good Z, Spitzer MH, Davis KL, Nolan GP. Automated mapping of phenotype space with single-cell data. Nat Methods. 2016;13(6):493–6. doi: 10.1038/nmeth.3863 27183440 PMC4896314

[9] Hauchamps P, Delandre S, Temmerman ST, Lin D, Gatto L. Visual Quality Control With CytoMDS, a Bioconductor Package for Low Dimensional Representation of Cytometry Sample Distances. Cytometry A. 2025;107(3):177–86. doi: 10.1002/cyto.a.24921 40035132

[10] Gachon E, Bigot J, Cazelles E, Bidet A, Vial J, Dumas P, et al. Low Dimensional Representation of Multi-Patient Flow Cytometry Datasets Using Optimal Transport for Measurable Residual Disease Detection in Leukemia. Cytometry Pt A. 2025;107(2):126–39. doi: 10.1002/cyto.a.24918 40028809

[11] Orlova DY, Zimmerman N, Meehan S, Meehan C, Waters J, Ghosn EEB, et al. Earth Mover’s Distance (EMD): A True Metric for Comparing Biomarker Expression Levels in Cell Populations. PLoS One. 2016;11(3):e0151859. doi: 10.1371/journal.pone.0151859 27008164 PMC4805242

[12] Mathew D, Giles JR, Baxter AE, Oldridge DA, Greenplate AR, Wu JE, et al. Deep immune profiling of COVID-19 patients reveals distinct immunotypes with therapeutic implications. Science. 2020;369(6508):eabc8511. doi: 10.1126/science.abc8511 32669297 PMC7402624

[13] Del Barrio E, Inouzhe H, Loubes J-M, Matrán C, Mayo-Íscar A. optimalFlow: optimal transport approach to flow cytometry gating and population matching. BMC Bioinformatics. 2020;21(1):479. doi: 10.1186/s12859-020-03795-w 33109072 PMC7590740

[14] Freulon P, Bigot J, Hejblum BP. CytOpT: Optimal transport with domain adaptation for interpreting flow cytometry data. Ann Appl Stat. 2023;17(2). doi: 10.1214/22-aoas1660

[15] Mukherjee S, Wethington D, Dey TK, Das J. Determining clinically relevant features in cytometry data using persistent homology. PLoS Comput Biol. 2022;18(3):e1009931. doi: 10.1371/journal.pcbi.1009931 35312683 PMC9009779

[16] Cuturi M. Sinkhorn distances: lightspeed computation of optimal transport. Advances in Neural Information Processing Systems. 2013;26.

[17] Dietz MV, Quintelier KLA, van Kooten JP, de Boer NL, Vink M, Brandt-Kerkhof ARM, et al. Adjuvant dendritic cell-based immunotherapy after cytoreductive surgery and hyperthermic intraperitoneal chemotherapy in patients with malignant peritoneal mesothelioma: a phase II clinical trial. J Immunother Cancer. 2023;11(8):e007070. doi: 10.1136/jitc-2023-007070 37536940 PMC10401259

[18] Emmaneel A, Quintelier K, Sichien D, Rybakowska P, Marañón C, Alarcón-Riquelme ME, et al. PeacoQC: Peak-based selection of high quality cytometry data. Cytometry A. 2022;101(4):325–38. doi: 10.1002/cyto.a.24501 34549881 PMC9293479

[19] Becton, Dickinson and Company. FlowJo Software. https://www.flowjo.com/ 2023.

[20] Quintelier KLA, Willemsen M, Bosteels V, Aerts JGJV, Saeys Y, Van Gassen S. CytoNorm 2.0: A flexible normalization framework for cytometry data without requiring dedicated controls. Cytometry Pt A. 2025;107(2):69–87. doi: 10.1002/cyto.a.24910 39871681

[21] Aghaeepour N, Finak G, FlowCAP Consortium, DREAM Consortium, Hoos H, Mosmann TR, et al. Critical assessment of automated flow cytometry data analysis techniques. Nat Methods. 2013;10(3):228–38. doi: 10.1038/nmeth.2365 23396282 PMC3906045

[22] Lux M, Brinkman RR, Chauve C, Laing A, Lorenc A, Abeler-Dörner L, et al. flowLearn: fast and precise identification and quality checking of cell populations in flow cytometry. Bioinformatics. 2018;34(13):2245–53. doi: 10.1093/bioinformatics/bty082 29462241 PMC6022609

[23] "Ester M, Kriegel HP, Sander J, Xu X. A density-based algorithm for discovering clusters in large spatial databases with noise. In: KDD, 1996. 226–31.

[24] Ye X, Ho JWK. Ultrafast clustering of single-cell flow cytometry data using FlowGrid. BMC Syst Biol. 2019;13(Suppl 2):35. doi: 10.1186/s12918-019-0690-2 30953498 PMC6449887

[25] Abe K, Minoura K, Maeda Y, Nishikawa H, Shimamura T. Model-based clustering for flow and mass cytometry data with clinical information. BMC Bioinformatics. 2020;21(Suppl 13):393. doi: 10.1186/s12859-020-03671-7 32938365 PMC7495858

[26] Hamming RW. Error Detecting and Error Correcting Codes. Bell System Technical Journal. 1950;29(2):147–60. doi: 10.1002/j.1538-7305.1950.tb00463.x

[27] Wickham H. ggplot2: Elegant Graphics for Data Analysis. New York: Springer-Verlag. 2016.

[28] Csárdi G, Nepusz T, Traag V, Horvát S, Zanini F, Noom D. igraph: Network analysis and visualization in R. 2025. 10.5281/zenodo.7682609 PMC13436765 42550872

[29] Kamada T, Kawai S. An algorithm for drawing general undirected graphs. Information Processing Letters. 1989;31(1):7–15. doi: 10.1016/0020-0190(89)90102-6

[30] "Hagberg AA, Schult DA, Swart PJ. Exploring Network Structure, Dynamics, and Function using NetworkX. In: Proceedings of the 7th Python in Science Conference, Pasadena, CA USA, 2008. 11–5.

[31] "Abu-Aisheh Z, Raveaux R, Ramel J-Y, Martineau P. An Exact Graph Edit Distance Algorithm for Solving Pattern Recognition Problems. In: Proceedings of the International Conference on Pattern Recognition Applications and Methods, 2015. 271–8. 10.5220/0005209202710278

[32] NIAID Visual & Medical Arts. Syringe. NIAID NIH BioArt Source. https://bioart.niaid.nih.gov/bioart/505 2024. 2026 April 24.

[33] NIAID Visual & Medical Arts. BDBlood Vial. https://bioart.niaid.nih.gov/bioart/52 2024. 2026 April 24.

[34] Diggins KE, Gandelman JS, Roe CE, Irish JM. Generating Quantitative Cell Identity Labels with Marker Enrichment Modeling (MEM). Curr Protoc Cytom. 2018;83:10.21.1-10.21.28. doi: 10.1002/cpcy.34 29345329 PMC5774653

