# Supplementary material for: CARGO: A Cytometry Analysis framework via Regularized Graph Optimal-transport
Source: PLoS Comput Biol. 2026 Jun 23;22(6):e1014358. doi: 10.1371/journal.pcbi.1014358 (PMC13313376; doi:10.1371/journal.pcbi.1014358)
Supplement: S1 Text — (PDF) [file pcbi.1014358.s001.pdf]

S1 Text

The following material is from our previous work on the optimal transport framework [1].

Optimal transport framework

The *transport problem* distributes a certain amount of *mass* from a set of sources to a set of destinations at minimum cost. There are two major factors in a transport problem: the *cost function* and the *transportation plan*. The cost function defines a fixed, non-negative effort required to transport unit mass from a source to a destination. This cost may only depend on the distance between the source and the destination or on other additional factors; in the former case, a Euclidean distance matrix between the sources and the destinations is a reasonable representation of effort.

Once the cost of transportation is represented, the remaining part of the problem involves transporting a non-negative amount of mass between sources and destinations, as described by a transportation plan. Various transportation plans result in different total costs, and the *optimal transport (OT) problem* aims to minimize this cost. The OT problem is *balanced* if the total mass at the sources equals the total mass at the destinations, and *unbalanced* otherwise [2].

Let r and c be two d dimensional vectors representing the amount of mass at the sources and the destinations, respectively. The number of sources and destinations could differ, but they can be considered equal without loss of generality. Let $U(r,c)$ be the set of all non-negative $d \times d$ matrices with row and column summing to r and c, respectively. Any matrix $P \in U(r,c)$ describes a transportation plan that transports the mass in r to c. Given a $d \times d$ cost matrix M, the total cost of mapping r to c using the transportation plan P is $\sum_{i,j} P_{ij} M_{ij}$. Thus the OT problem between r and c given cost M can be formulated by Equation 1, where $D_M(r,c)$ is the optimal transport distance:

$$\begin{gathered} D_M(r,c) = \min_{P \in U(r,c)} \sum_{i,j} P_{ij} M_{ij}, \\ \text{subject to} \quad \sum_j P_{ij} = r_i, \quad \sum_i P_{ij} = c_j, \quad P_{ij} \geq 0, \quad \forall i,j \leq d. \end{gathered} \tag{1}$$

The masses in r and c could be normalized to sum to one, and then both r and c can be interpreted as probability distributions.

For $D_M(r,c)$ to be a metric, the cost matrix M has to be a metric matrix [3, 4, 5] satisfying the conditions shown in Equation 2.

$$\begin{gathered} \text{Non-negativity: } M_{ij} \geq 0, \\ \text{Identity: } M_{ii} = 0, \\ \text{Symmetry: } M_{ij} = M_{ji}, \\ \text{Triangle inequality: } M_{ij} \leq M_{ik} + M_{kj}, \quad \forall i,j,k \leq d. \end{gathered} \tag{2}$$

The OT is a convex optimization problem that can be solved using various approaches [6, 7]. For a general cost matrix, the computational cost scales as $\mathcal{O}(d^3 \log(d))$ [8], which prevents scaling the solution to large problem sizes. Earlier approximate solutions obtained by putting constraints on the cost matrix could result in a loss of applicability and performance [9]. A later approximation to the original OT problem using an entropic regularization scheme was proposed by [10] to reduce the computational complexity. The scheme employs the Sinkhorn-Knopp matrix scaling algorithm [11, 12], and hence the name *Sinkhorn distance* for its objective function.

Sinkhorn distance

A straightforward way of thinking about a transportation plan is by noticing that if a source contains more mass, it should originate more, and if a destination requires more mass, it should receive proportionally more. Such a transportation plan is represented by rc^T, and the optimal plan P should be somewhere around the distribution rc^T. Simply speaking, the idea of the entropic regularization scheme by [10] is to choose P from a smaller set near rc^T, instead of the entire set $U(r,c)$.

To capture these ideas, [10] imposes an additional constraint of Kullback-Leibler (KL) divergence on the OT formulation, as shown in Equation 3, and computes the Sinkhorn distance $D^*_{M,\alpha}(r,c)$. This constraint introduces a set $U_\alpha(r,c) \subset U(r,c)$ from which an optimal transportation plan P is selected. The KL divergence distance between P and rc^T is set to be smaller than a predefined parameter α. In other words, P should belong to a distribution near rc^T.

$$\begin{gathered} D^*_{M,\alpha}(r,c) = \min_{P \in U_\alpha(r,c)} \sum_{i,j} P_{ij} M_{ij}, \\ \text{subject to} \quad \mathbf{KL}(P|rc^T) \leq \alpha, \quad \sum_j P_{ij} = r_i, \quad \sum_i P_{ij} = c_j, \quad \forall i,j \leq d. \end{gathered} \tag{3}$$

The entropy (h) of the transportation plan (P) and the mass vectors (r and c) are given in Equation 4:

$$\begin{gathered} h(P) = -\sum_{ij} P_{ij} \log P_{ij}, \\ h(r) = -\sum_i r_i \log r_i, \quad h(c) = -\sum_j c_j \log c_j. \end{gathered} \tag{4}$$

We proceed to express the KL divergence constraint in terms of the entropy:

$$\begin{aligned} \mathbf{KL}(P|rc^T) &= \sum_{ij} P_{ij} \log \frac{P_{ij}}{r_i c_j} \\ &= \sum_{ij} P_{ij} \log P_{ij} - \sum_{ij} P_{ij} \log r_i - \sum_{ij} P_{ij} \log c_j \\ &= \sum_{ij} P_{ij} \log P_{ij} - \sum_i r_i \log r_i - \sum_j c_j \log c_j \quad [\because \sum_j P_{ij} = r_i, \sum_i P_{ij} = c_j] \\ &= -h(P) + h(r) + h(c) \leq \alpha. \end{aligned} \tag{5}$$

Thus, the new constraint states that the entropy of P should be large enough to satisfy

$$h(P) \geq h(r) + h(c) - \alpha,$$

which constrains P to be chosen from the Kullback-Leibler ball of level α centered about rc^T (see Fig 1 in [10]).

This interpretation makes the OT problem non-convex, and an alternative formulation of Sinkhorn distance is required for ease of optimization. For every pair (r,c), each α corresponds to a Lagrange multiplier $\lambda \in [0,\infty)$ such that $D^*_{M,\alpha}(r,c) = D^\lambda_M(r,c)$. The distance D^λ_M, shown in Equation 6, is called the dual-Sinkhorn divergence by [10].

$$\begin{gathered} D^\lambda_M(r,c) = \sum_{i,j} P^\lambda_{ij} M_{ij}, \quad \text{where } P^\lambda = \operatorname*{argmin}_{P \in U(r,c)} \sum_{i,j} P_{ij} M_{ij} - \lambda h(P), \\ \text{subject to} \quad \sum_j P_{ij} = r_i, \quad \sum_i P_{ij} = c_j, \quad \forall i,j \leq d. \end{gathered} \tag{6}$$

By introducing two dual variables ϕ and ψ for each of the two equality constraints of Equation 6, the Lagrangian of the objective function can be written as Equation 7.

$$\mathcal{L}(P,\phi,\psi) = \sum_{i,j} P_{ij} M_{ij} - \lambda h(P) + \sum_i \phi_i (\sum_j P_{ij} - r_i) + \sum_j \psi_j (\sum_i P_{ij} - c_j). \tag{7}$$

The derivative of the Lagrangian objective function with respect to P_{ij}, for any pair (i,j), can be set to zero to obtain an extremum; the second derivative of the Lagrangian, $(\frac{\lambda}{P_{ij}})$, is positive since both the

numerator and the denominator are positive, and thus we have obtained a minimizer of the Lagrangian.

$$\begin{aligned} \frac{\partial \mathcal{L}}{\partial P_{ij}} &= M_{ij} + \lambda + \lambda \log P_{ij} + \phi_i + \psi_j = 0. \\ \implies \quad P_{ij} &= e^{-\frac{\phi_i}{\lambda} - \frac{1}{2}} . e^{-\frac{M_{ij}}{\lambda}} . e^{-\frac{\psi_j}{\lambda} - \frac{1}{2}} \\ &\equiv u_i K_{ij} v_j \quad [u_i = e^{-\frac{\phi_i}{\lambda} - \frac{1}{2}}, v_j = e^{-\frac{\psi_j}{\lambda} - \frac{1}{2}}, K = e^{-\frac{M}{\lambda}}] \end{aligned} \tag{8}$$

Given K, r and c, the Sinkhorn-Knopp matrix scaling algorithm converges to a solution P^λ of the following form:

$$\exists u, v : P^\lambda = \mathbf{diag}(u) K \mathbf{diag}(v). \tag{9}$$

P^λ should have the correct row and column sums, as shown in Equation 6. We deduce the update rule for the Sinkhorn-Knopp algorithms from those constraints in the following manner:

$$\begin{aligned} &\sum_j P^\lambda_{ij} = r_i, && \sum_i P^\lambda_{ij} = c_j \\ \implies &\sum_j u_i K_{ij} v_j = r_i \quad \text{[Equation 8]} & \implies &\sum_i u_i K_{ij} v_j = c_j \quad \text{[Equation 8]} \\ \implies &u_i \sum_j K_{ij} v_j = r_i & \implies &v_j \sum_i u_i K_{ij} = c_j \\ \implies &u_i = r_i / \sum_j K_{ij} v_j & \implies &v_j = c_j / \sum_i u_i K_{ij} \end{aligned}$$

Thus the update rule for the Sinkhorn-Knopp algorithm can be written as Equation 10, where v can be initialized randomly.

$$\begin{aligned} u &= r./(Kv), \\ v &= c./(K^T u). \end{aligned} \tag{10}$$

[10] observes that the number of iterations in the Sinkhorn-Knopp algorithm is bounded independent of d. Thus, the cost of computing D^λ_M is $\mathcal{O}(d^2)$, which is an improvement over $\mathcal{O}(d^3 \log(d))$. [10] describes an approach to compute the Sinkhorn distance ($D^*_{M,\alpha}(r, c)$) through the dual-Sinkhorn divergence ($D^\lambda_M(r, c)$), and also reports that the dual-Sinkhorn divergence does not perform worse than the classic optimal transport distances. Therefore, we use the dual-Sinkhorn divergence to measure the distance between the cell populations in our experiments and refer to as the Sinkhorn distance. We utilize the *POT Python Optimal Transport* library for computing the dual-Sinkhorn divergences [13].

GED trajectory sensitivity analysis

To assess the robustness of the graph edit distance formulation to the relative weighting of population proportions and phenotypic relationships, we performed a sensitivity analysis across a range of vertex and edge edit-cost weights. Vertex attributes represent cell-population proportions and were scaled to the interval [0,1], whereas edge attributes encode inter-population phenotypic dissimilarities quantified by Sinkhorn distances. To control for sample-specific scaling effects and emphasize relative phenotypic structure, Sinkhorn distance matrices were L_1-normalized within each graph before GED computation.

In the GED formulation, a single vertex edit weight w_V was applied uniformly to all vertex edit operations (substitution, insertion, and deletion), and a single edge edit weight w_E was applied uniformly to all edge edit operations. In the main text, results are reported for $w_V = w_E = 1$. To evaluate sensitivity to these parameters, we additionally considered the following combinations:

$$(w_V, w_E) \in \{(0.25, 1), (0.5, 1), (0.75, 1), (1, 0.75), (1, 0.5), (1, 0.25)\}.$$

For each weight pair, GED trajectories were computed for all patients across the longitudinal time points (baseline, post-first vaccination, and post-third vaccination). When the edge edit-cost weight w_E was varied while keeping $w_V = 1$, only minor changes in the GED trajectories were observed (see S1 Fig AA, S1 Fig

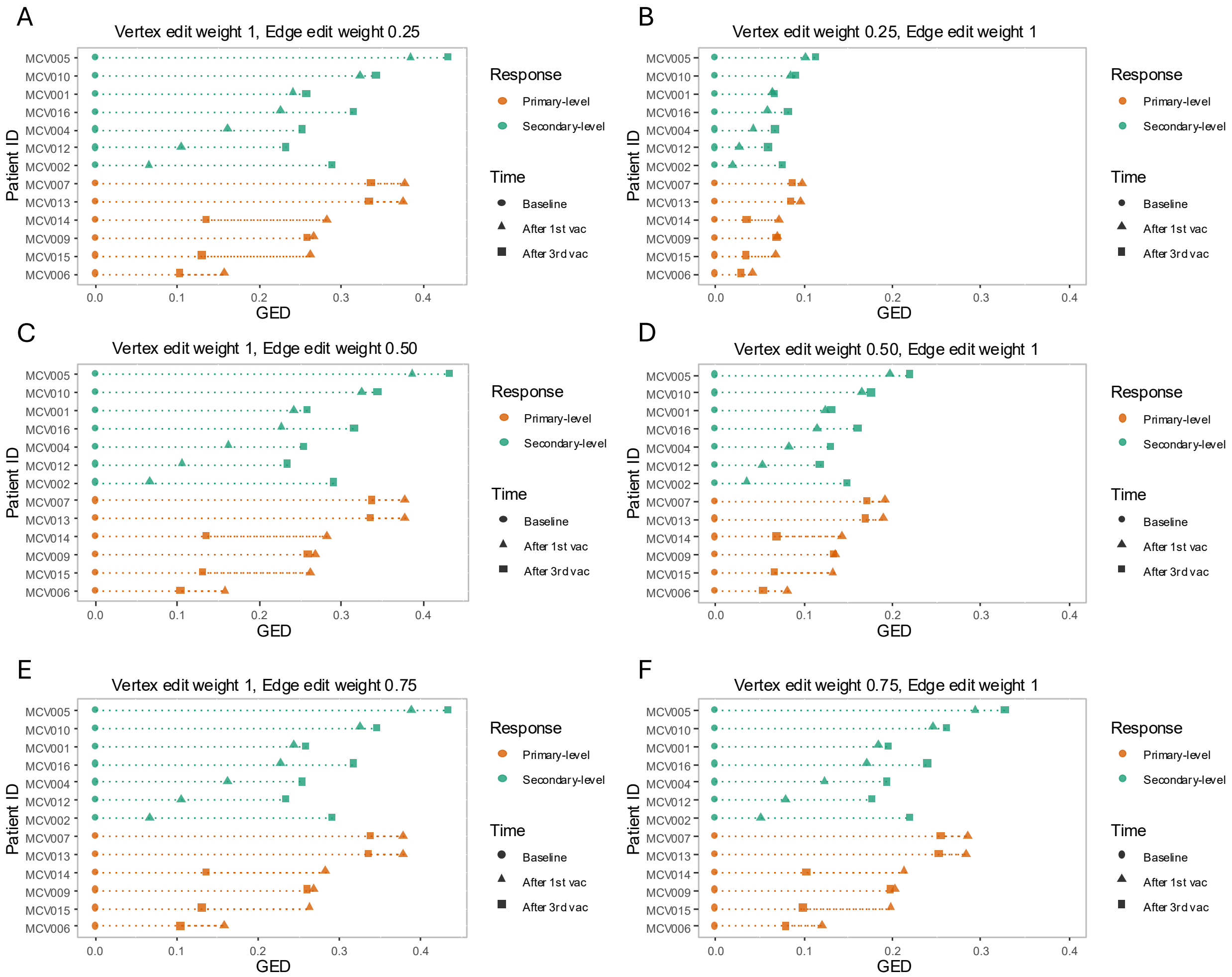

Fig A: Sensitivity of GED trajectories to vertex and edge edit cost weighting. The GED trajectories are computed for all patients across baseline, post-first vaccination, and post-third vaccination time points under varying vertex (w_V) and edge (w_E) edit cost weights. (A, C, E) GED trajectories obtained by varying the edge edit cost weight ($w_E = \{0.25, 0.5, 0.75\}$) while keeping the vertex edit cost fixed ($w_V = 1$), resulting in only minor changes to the absolute GED values. (B, D, F) GED trajectories obtained by varying the vertex edit cost weight ($w_V = \{0.25, 0.5, 0.75\}$) while fixing the edge edit cost ($w_E = 1$), which alters the absolute GED scale while preserving trajectory shape and relative sample ordering.

AC, S1 Fig AE). This behavior is expected because individual L_1-normalized edge weights are substantially smaller than the vertex attributes, resulting in a smaller contribution of edge edit operations to the total GED. This scale difference is a direct consequence of the chosen normalization and does not imply that phenotypic structure is ignored; rather, edge edits primarily influence the relative ordering and shape of the trajectories rather than the absolute GED magnitude. Conversely, varying w_V while fixing $w_E = 1$ changed the absolute GED values but preserved the overall trajectory shape and the relative ordering of samples across time (see S1 Fig AB, S1 Fig AD, S1 Fig AF).

Importantly, patient stratification into primary and secondary-level responders based on GED trajectories remained unchanged across all tested weight combinations (S1 Fig A). These results indicate that while the absolute scale of the GED reflects the relative emphasis placed on population proportion versus phenotypic relationships, the longitudinal trends and downstream biological interpretations are robust to reasonable variations in vertex and edge edit cost weights.

Computational parameters

- **Entropic regularizer parameter, λ:** For both MPM and AML datasets, we used $\lambda = 0.1$ to compute the pair-wise Sinkhorn distance between the cell populations. We chose *numItermax* $= 2000$ (which is doubled than the default) and *stopThr* $= 10^{-9}$ (error) in the function call *ot.sinkhorn()* from the *POT Python library.*
- **Weights of vertex and edge edit:** For the reported results, we used $w_V = 1$ as the weight of vertex edits and $w_E = 1$ as the weight of edge edits. For the sensitivity analysis of the graph edit distance trajectory (on MPM dataset), we used the following pairs of weights:

$$(w_V, w_E) \in \{(0.25, 1), (0.5, 1), (0.75, 1), (1, 0.75), (1, 0.5), (1, 0.25)\}.$$

- **Phenotype description of cell populations:** The phenotype descriptions are annotated using immunological knowledge.
  - **MPM dataset (13 cell populations)**
    - **NK cell:** $CD3^-$ $CD56^+$ $CD8^+$ $CD4^-$ $FoxP3^-$ $CCR7^{low}$ $CD45RA^+$
    - **NKT cell:** $CD3^+$ $CD56^+$ $CD8^+$ $CD4^-$ $FoxP3^-$ $CCR7^{low}$ $CD45RA^{low}$
    - **$CD4^+$ Treg:** $CD3^+$ $CD56^-$ $CD8^-$ $CD4^+$ $FoxP3^+$ $CCR7^+$ $CD45RA^+$
    - **$CD4^+$ EM:** $CD3^+$ $CD56^-$ $CD8^-$ $CD4^+$ $FoxP3^-$ $CCR7^-$ $CD45RA^-$
    - **$CD4^+$ EMRA:** $CD3^+$ $CD56^-$ $CD8^-$ $CD4^+$ $FoxP3^-$ $CCR7^-$ $CD45RA^+$
    - **$CD4^+$ CM:** $CD3^+$ $CD56^-$ $CD8^-$ $CD4^+$ $FoxP3^-$ $CCR7^+$ $CD45RA^-$
    - **$CD4^+$ Naïve T-cell:** $CD3^+$ $CD56^-$ $CD8^-$ $CD4^+$ $FoxP3^-$ $CCR7^+$ $CD45RA^+$
    - **$CD8^+$ EM:** $CD3^+$ $CD56^-$ $CD8^+$ $CD4^-$ $FoxP3^-$ $CCR7^-$ $CD45RA^-$
    - **$CD8^+$ EMRA:** $CD3^+$ $CD56^-$ $CD8^+$ $CD4^-$ $FoxP3^-$ $CCR7^-$ $CD45RA^+$
    - **$CD8^+$ CM:** $CD3^+$ $CD56^-$ $CD8^+$ $CD4^-$ $FoxP3^-$ $CCR7^+$ $CD45RA^-$
    - **$CD8^+$ Naïve T-cell:** $CD3^+$ $CD56^-$ $CD8^+$ $CD4^-$ $FoxP3^-$ $CCR7^+$ $CD45RA^+$
    - **Other $CD3^-$:** $CD3^-$ $CD56^+$ $CD8^{low}$ $CD4^-$ $FoxP3^-$ $CCR7^{low}$ $CD45RA^+$
    - **Other $CD3^+$:** $CD3^+$ $CD56^{low}$ $CD8^+$ $CD4^{low}$ $FoxP3^+$ $CCR7^+$ $CD45RA^+$
  - **AML dataset (4 cell populations)**
    - **AML Blasts:** $HLA\text{-}DR^+$ $CD117^+$ $CD45^-$ $CD34^+$ $CD38^+$
    - **Monocytes:** $HLA\text{-}DR^+$ $CD117^-$ $CD45^+$ $CD34^-$ $CD38^-$
    - **Lymphocytes:** $HLA\text{-}DR^{dc}$ $CD117^-$ $CD45^+$ $CD34^-$ $CD38^-$
    - **Unclassified:** $HLA\text{-}DR^{dc}$ $CD117^{dc}$ $CD45^{dc}$ $CD34^{dc}$ $CD38^{dc}$
- **Adjacency of graph topology:** For the AML dataset, we used the minimum spanning tree (MST) as the graph topology. For the MPM dataset, we used the following expert-curated layout.
  - **NK cell** → Other $CD3^-$, NKT cell
  - **NKT cell** → Other $CD3^+$, NK cell
  - **$CD4^+$ Treg** → $CD4^+$ Naïve T-cell
  - **$CD4^+$ EM** → $CD4^+$ Naïve T-cell
  - **$CD4^+$ EMRA** → $CD4^+$ Naïve T-cell
  - **$CD4^+$ CM** → $CD4^+$ Naïve T-cell
  - **$CD4^+$ Naïve T-cell** → Other $CD3^+$, $CD4^+$ Treg, $CD4^+$ EM, $CD4^+$ EMRA, $CD4^+$ CM
  - **$CD8^+$ EM** → $CD8^+$ Naïve T-cell
  - **$CD8^+$ EMRA** → $CD8^+$ Naïve T-cell
  - **$CD8^+$ CM** → $CD8^+$ Naïve T-cell
  - **$CD8^+$ Naïve T-cell** → Other $CD3^+$, $CD8^+$ EM, $CD8^+$ EMRA, $CD8^+$ CM
  - **Other $CD3^-$** → NK cell
  - **Other $CD3^+$** → NKT cell, $CD4^+$ Naïve T-cell, $CD8^+$ Naïve T-cell

References

[1] Shemonti AS, Plebani E, Biscola NP, Jaffey DM, Havton LA, Keast JR, et al. A novel statistical methodology for quantifying the spatial arrangements of axons in peripheral nerves. Frontiers in Neuroscience. 2023;17:1072779.

[2] Peyré G, Cuturi M. Computational Optimal Transport: With Applications to Data Science. Foundations and Trends in Machine Learning. 2019 Feb;11(5-6):355-607.

[3] Villani C. The Wasserstein distances. In: Villani C, editor. Optimal Transport: Old and New. Berlin, Heidelberg: Springer; 2009. p. 93-111.

[4] Avis D. On the Extreme Rays of the Metric Cone. Canadian Journal of Mathematics. 1980 Feb;32(1):126-44.

[5] Brickell J, Dhillon IS, Sra S, Tropp JA. The Metric Nearness Problem. SIAM Journal on Matrix Analysis and Applications. 2008 Jan;30(1):375-96.

[6] Ahuja RK, Magnanti TL, Orlin JB. Network Flows - Theory, Algorithms and Applications. Prentice Hall; 1993.

[7] Orlin JB. A Faster Strongly Polynomial Minimum Cost Flow Algorithm. Operations Research. 1993 Apr;41(2):338-50.

[8] Pele O, Werman M. Fast and robust Earth Mover's Distances. In: 2009 IEEE 12th International Conference on Computer Vision; 2009. p. 460-7. ISSN: 2380-7504.

[9] Grauman K, Darrell T. Fast Contour Matching Using Approximate Earth Mover's Distance. In: Proceedings of the 2004 IEEE Computer Society Conference on Computer Vision and Pattern Recognition, 2004. CVPR 2004.. vol. 1; 2004. p. 1-8.

[10] Cuturi M. Sinkhorn distances: Lightspeed computation of optimal transport. Advances in Neural Information Processing Systems. 2013;26.

[11] Sinkhorn R, Knopp P. Concerning nonnegative matrices and doubly stochastic matrices. Pacific Journal of Mathematics. 1967 May;21(2):343-8.

[12] Knight PA. The Sinkhorn–Knopp Algorithm: Convergence and Applications. SIAM Journal on Matrix Analysis and Applications. 2008 Jan;30(1):261-75.

[13] Flamary R, Courty N, Gramfort A, Alaya MZ, Boisbunon A, Chambon S, et al. POT: Python Optimal Transport. Journal of Machine Learning Research. 2021;22(78):1-8.
